# Investigation of Menstrual Hygiene and Self-Care Skills of Adolescent Girls with Autism Spectrum Disorder: Mother Views

**DOI:** 10.1007/s10803-024-06446-8

**Published:** 2024-07-02

**Authors:** Rukiye Arslan, Derya Yanık, Raziye Pekşen Akça

**Affiliations:** 1 Department of Child Development, Batman University Faculty of Health Sciences, Batman Merkez, Türkiye; 2 Faculty of Health Sciences, Department of Nursing, Batman University, Batman Merkez, Türkiye; 3 Develi Huseyin Şahin Vocational College Child Care and Youth Services, Kayseri University, Kayseri, Türkiye

**Keywords:** Adolescent, Autism spectrum disorder, Menstrual hygiene, Self-care

## Abstract

**Purpose:**

Puberty a period of transition from childhood to adulthood, poses problems that are difficult to manage for typically developing adolescents, but even more difficult for adolescents with autism. It is vital that girls with autism spectrum disorder (ASD), like their typically developing peers, are able to manage these physiological processes in a healthy way and learn self-care and hygiene behaviours without being dependent on others. Given the contribution of mothers to the menstrual hygiene and self-care skills of adolescent girls, this study aims to explore the views of mothers.

**Methods:**

The study is a case study designed in the qualitative research model. The research was carried out with the participation of 10 mothers met the necessary criteria and agreed to take part in the study voluntarily. The data obtained were subjected to descriptive and thematic analysis. Codes, sub-themes and main themes were created.

**Results:**

The research identifies the themes of self-care and menstrual hygiene, preparations made during menstruation, behavioral problems in adolescents, the person who carries out hygiene and self-care, and situations that tire/strain mothers during menstruation.

**Conclusion:**

At the end of the research, it was concluded that the adolescent girls with ASD are mostly unable to perform their self-care and hygiene adequately, and that the mothers do nothing to prepare their daughters for adolescence. It was also concluded that the most stressful situation for mothers during adolescence is usually the difficulty their daughters have in using sanitary pads, cleaning armpits and genital hair, and bathing.

## Introduction

Autism Spectrum Disorder (ASD) is a neuro-developmental disorder, the symptoms of which appear between the ages of 0–3. And according to DSM-5 (Diagnostic and Statistical Manual of Mental Disorders), this disorder shows characteristic features such as social inadequacy in social interaction, delayed or missing verbal and nonverbal communication skills, restricted interest fields and repeated behaviours (Corona et al., 2016; Arslan & Sağlam, 2021). ASD is seen less in females compared to males. According to the US epidemiological predictions, it is stated that the clinically reported male-female ratio is 1 female adolescent for every 4.3 male diagnosed with ASD (Navot et al., 2017). However, recent studies suggest that this ratio comes close to 3:1 (Loomes et al., 2017). When looked into it clinically, it is seen that the girls with ASD exhibit less problematic behaviours (Cridland et al., 2014) but more social problems than boys (Navot et al., 2017); in addition, the girls are able to camouflage ASD symptoms better (Corbett et al., 2020).

Adolescence, considered the transition period from childhood to adulthood, is one of the most significant developmental periods during which individuals undergo physical, cognitive, social and emotional changes. While the current characteristics of this period cause problems that are difficult to manage even for typically developing teenagers, when combined with the characteristic features of ASD, teenagers with autism may experience different or amplified challenges associated with puberty (Corona et al., 2016; Visser et al., 2017). In the normal developmental process, individuals receive information about adapting to the adolescent period mainly from their friends, family members and school environment; however, individuals with ASD have difficulties in participating in a friend group due to inadequacies in social interaction and communication; therefore, they are exposed to difficulties in adapting to this period (Erbaş & Kurt, 2022).The physiological, emotional and social changes experienced by individuals with ASD during adolescence cause anxiety for both individuals and families, yet parents do not have enough information about how to deal with these issues during this time and how to prepare their children for this process (Cummins et al., 2020; Mademtzi et al., 2018; Güven, 2021).

One of the most significant changes of the adolescent period in girls is the start of the menstrual cycle, and the self-care specific to this period is expected to be managed by the individual independently (Bitsika & Sharpley, 2018; Gönenç et al., 2020). However, it is noted that the adolescent girls with ASD have difficulties in becoming aware of the fact that menstruation has started and that perineal cleansing, hand hygiene and pad use are necessary (Gönenç et al., 2020). Studies of adolescent girls with ASD have found that mothers are particularly concerned about menstruation and hygiene (Cridland et al., 2014; Navot et al., 2017; Quint et al., 2016) and that parents request medical help to suppress or eliminate menstruation (Cummins et al., 2020).

Each adolescent girl with ASD has needs specific to her because each of them has variable characteristics and different degrees of sensory and perceptual difficulties. Therefore, it is quite important to start the education of adolescent girls with ASD as early as possible (Özgür, 2013; Fazlıoğlu & Yurdakul, 2007; Arslan & Pekşen Akça, 2022). Before the menstruation period of teenage girls start, it is necessary that they should be prepared for this process, and education and feedback about the physical changes in their bodies during puberty should continue so that they can learn the monthly cycle (Koegel & LaZebnik, 2009). However, it should not be forgotten that developing the necessary hygiene skills and teaching them how to cope with menstruation period could be challenging due to language and communicational hardships (Gabriels & Van Bourgondien, 2007). In individuals with ASD, there are limited number of studies about changes during adolescent period and adapting to these changes. However, there are quite few studies especially about adolescent period of girls with ASD (Burke et al., 2010; Cummins et al., 2020; Gabriels & Van Bourgondien, 2007; Hamilton et al., 2011; Henault, 2006; Klett & Turan, 2012). Therefore, this study was carried out based on the mothers’ views to investigate menstrual hygiene and self-care skills of adolescent girls diagnosed with ASD and to provide contribution for the literature in this respect. For this purpose, the hypotheses of the study are stated below:

### Hypothesis 1

‘Adolescent girls with ASD have difficulties with menstrual hygiene during the menstrual period’.

### Hypothesis 2

‘Adolescent girls with ASD have difficulties with self-care during the menstrual period’.

## Methods

### Research Model

In this case study, qualitative survey method was utilized. Qualitative survey method is a method where studies are carried out concentrating on phenomena, incidents or behaviours happening in natural environment, and is addressed with a holistic perspective (Büyüköztürk et al., 2016). On the other hand, a case study is a kind of investigation where only case or an event is examined in detail (Subaşı & Okumuş, 2017).

### Participants

The study group of the research was determined by using criterion sampling, which is one of the purposive sampling methods. The main purpose of criterion sampling is to select a sample that reflects the characteristics of the majority of the population. Furthermore, purposive sampling is a type of sampling that is used in studies that are formed according to certain criteria (Büyüköztürk et al., 2016; Sümer et al., 2007; Özen & Gül, 2007). The inclusion criteria for this research are Being a mother, having a girl with ASD who is in puberty, voluntarily participating in the study and completing the form completely. Exclusion criteria from the study are defined as the participant’s request to withdraw from the study at any stage and completing the form incompletely. This study was conducted in a rural city in the Southeastern Anatolia Region of Turkey. Although the city has high cultural diversity, its economic and social development level is low. Permission to collect data for the study was obtained from the Batman Provincial Directorate of National Education. Later, through the Provincial Directorate of National Education, the contact information of mothers of adolescent autistic girls attending private educational institutions and special education subclasses affiliated to public schools was accessed. Telephone interviews were conducted with these mothers and they were invited to participate in the study. As a result, 15 mothers of adolescent girls with ASD were interviewed. As a result of these interviews, face-to-face interviews were conducted with the mothers who agreed to participate in the study. Five mothers were excluded from the study because one of them did not want to participate in the study and the other 4 were not included in the study because their daughters had not yet started menstruation. The demographic information of the mothers who participated in the study is shown in Table 1 as frequencies (F) and percentages (%).

**Table 1:** Demographic information of study group

		F	%
Teenager’s Age	11 years old	3	30
	12 years old	3	30
	13 years old	1	10
	14 years old	2	20
	15 years old	1	10
Age when teenager started menstruation	11 years old	3	30
	12 years old	5	50
	13 years old	2	20
Education status of mother	Illiterate	6	60
	Primary school	2	20
	Secondary school	-	-
	High school	1	10
	University	1	10
Age of mother	30–39	7	70
	40–49	1	10
	50 and over	2	20
Does mother work in a job with income?	Yes	1	10
	No	9	90
Income level of family	Less income than expenses	7	70
	Income equal to expenses	2	20
	Income more than expenses	1	10
Number of children	2 children	1	10
	3 children	2	20
	4 children	4	40
	5 and more	3	30
Did your daughter start to menstruate regularly?	Yes	10	100
	No	-	-
If “Yes”, age when teenager started menstruated	11 years old	3	30
	12 years old	5	50
	13 years old	2	20

Looking at Table 1, it was found that 30% of the adolescent girls participating in the study were between the ages of 11 and 12, and that 50% of them started menstruating at the age of 12. It was reported that 60% of the mothers were illiterate; 70% of the mothers were between the ages of 30–39; and 90% of the mothers did not work in any income-generating occupation. It was found that the income of 70% of the participants was less than their expenses and that 40% of the families had 4 children. It was found that 100% of girls with ASD started menstruating and 50% of them started menstruating for the first time at the age of 12.

### Data Collecting Tools

In the study, “Demographic Information Form”, Semi-Structured Interview Form” and “Open Ended Questions” prepared by the researchers as data collecting tools were used. The researchers prepared an interview form consisting of open-ended and semi-structured questions. Opinions of 7 specialists were received for this form. Out of these specialists, five of them have completed their PhDs in child development, one in gynaecology nursing and one in psychiatric nursing all of whom still work as academicians.

The interview form was finalised in accordance with the views of the experts. The demographic information form includes the following items: The age at which the adolescent girl started menstruating, the mother’s educational status, the mother’s age, the mother’s employment status, the family’s income level, the number of children in the family, and whether the girl menstruates regularly. While the questions related to self-care and menstrual hygiene are in the form of semi-structured interviews, the open-ended questions are as follows “What kind of preparations did/do you make for your daughter’s menstruation?”, “What are the positive and negative changes in your daughter’s behaviour during menstruation?”, “What kind of changes have happened in your daughter’s obsessive behaviour during menstruation? ”, “Who cleans and cares for your daughter during menstruation?”, “What has tired you the most during your daughter’s menstruation?”, “Have you explained to your daughter who to ask for help during menstruation when you are absent?”

### Collecting Data

The research data were collected via face-to-face interviews with the mothers after the ethical approval was obtained from the Scientific Ethics Committee of Batman University (dated 13.04.2022 and decision number 2022/04/06). After the mothers were informed about the study, the data were collected by means of interviews lasting approximately 25–30 min with those who voluntarily agreed to participate in the study.

### Analysis of the Data

In the analysis of the research data, descriptive analysis and content analysis techniques were used. Descriptive analysis is a technique that is generally used to analyse data without requiring a detailed analysis of the findings obtained in qualitative research (Baltacı, 2019). Accordingly, in the first stage of the analysis of the research data, questions about demographic information, self-care and menstrual hygiene were examined using the descriptive analysis technique (frequency and %).

Content analysis is a technique used to analyse some words of a text, student pictures, television programs and footage, or to analyse data such as interviews, discussions, conversations, etc., with coding based on certain rules. Content analysis is especially used in the analysis of data obtained from interviews and observations. In content analysis, concepts that are related to each other are first categorized and then analysed inductively (Büyüköztürk et al., 2016; Pope et al., 2006).

In this study, first, codes, (M1, M2, ………M10), were given to mothers of each adolescent girl with ASD. The answers that the mothers gave to the semi-structured questions were converted into written texts by the researchers. These texts were then reread and codes were generated. From the codes generated, sub-themes were identified and finally these sub-themes were summarised under the main themes. The Consolidated Criteria for Reporting Qualitative Research (COREQ) guidelines were followed in reporting the study data (Tong et al., 2007).

## Results

The results of the study were evaluated in two groups as quantitative and qualitative. While in the first group, quantitative data were analysed, in the second group, qualitative data were analysed.

### Data Obtained Through Scanning Method

When Table 2 is examined; 50% of the mothers participating in the study stated that their daughters could not independently go to the toilet and do her self-cleaning after the toilet independently. And 70% of the mothers, who participated in the study, stated that their daughters could not independently comb their hair and brush their teeth. Also 80% of the mothers stated that their daughters could not take a bath independently, 70% stated that their daughters could not dress or undress independently. Moreover, 90% of the mothers stated that their daughters could not cut their nails regularly. All mothers who participated in the study reported that they did not do any work/preparation to prepare their daughters for puberty. In addition, it was stated that 80% did not tell their daughters that their breasts would grow bigger, and 90% did not tell their daughters that hairs would grow in their legs, armpits and genital area. Also, 80% of the mothers who participated in the study stated that they did not inform their daughters that they could have menstrual blood flowing from their genital area / (vagina) and did not tell them what to do when the blood came, and did not teach them how to use sanitary pads.

**Table 2:** Semi-structured interview form

Questions about self-care and menstrual hygiene			
		F	%
Can your daughter do her toilet and perform cleaning after toilet independently?	Yes	5	50
	No	5	50
Can your daughter comb her hair independently?	Yes	3	30
	No	7	70
Can your daughter brush her teeth independently?	Yes	3	30
	No	7	70
Can your daughter bath independently?	Yes	2	20
	No	8	80
Can your daughter get dressed and undressed independently?	Yes	3	30
	No	7	70
Can your daughter trim her fingers regularly?	Yes	1	10
	No	9	90
Have you had any activity/preparation/talk to your daughter to prepare her adolescence before entering menstruation period?	Yes	-	-
	No	10	100
Have you benefited from any materials (pictures, videos, written documents, etc.) while informing her about menstruation?	Yes	-	-
	No	10	100
Has anybody else, apart from you, given your daughter any information about menstruation period?	Yes	-	-
	No	10	100
Have you ever mentioned your daughter about possible emotional changes during menstruation?	Yes	-	-
	No	10	100
Have you told your daughter about the physical changes that may occur in women during menstruation?	Yes	1	10
	No	9	90
Have you told your daughter that her breasts will grow bigger?	Yes	2	20
	No	8	80
Have you told your daughter that hairs will grow on her legs, armpits and genital areas?	Yes	1	10
	No	9	90
Have you taught your daughter how to clean her genital and armpit hairs?	Yes	-	-
	No	10	100
Have you informed your daughter about the menstrual period (menstruation, puberty, etc.)?	Yes	-	-
	No	10	100
Have you told your daughter that blood can come from her genital area (vagina), and what she should do when blood comes?	Yes	2	20
	No	8	80
Have you taught your daughter how to use sanitary pads?	Yes	2	20
	No	8	80

### Results About Qualitative Data

In this part, the data obtained from open-ended questions were given place in the results. Code “n” expresses the views of mothers about their daughter with ADS, and code “f” expresses the frequency how often they are used by the mothers, respectively. Table 3 shows the mothers’ views about preparations made for their daughters during their menstruation periods.

**Table 3:** Mothers’ views about preparations made for menstruation period of their daughters

Preparations made	*n*	f
Preparations for Self-Care		
No Preparations	8	8
Explaining the Physical Changes	2	2
Preparations for Seeking Support		
At the state to be able to ask for help	-	-
Not at the state to be able to ask for help	10	10
**Grand Total**	**20**	**20**

(n: number of responding participants, f: repetition number of the responds)

According to Table 3, when mothers’ views about preparations made for their daughters during menstruation period are examined, it is seen that they generally did not do any preparations and that their daughters were not in the manner to ask for help. Merely, 2 mothers stated that they mentioned their daughters about possible physical changes.

Quoted statements that can be examples of mothers’ views on the preparations made for their daughters during adolescence are given below:

*(M2) “…I did not have any preparations.”*

*(M3) “…I have no preparations; and I do not know what I should do.”*

*(M5) “…I told my daughter that her breasts will grow bigger, and she will have hairs in special areas.”*

*(M7) “…Whom are we to trust? Thankfully, I am alive. May God give me health and do not leave my daughter to anyone else’s care.”*

*(M8) “…I did not explain anything, yet even if I did, she wouldn’t understand.”*

According to Table 4, when the views of the mothers on the behavioural problems observed in their daughters during adolescence are examined, it is determined that they mostly observed an increase in their negative behaviour, and that there was an increase in obsessive actions and that their daughters became more aggressive. Only 1 mother stated that there was no change in her daughter’s behaviour. Quoted statements that can be examples of the views of mothers on behavioural problems observed in their daughters during adolescence are given below:

**Table 4:** Mothers’ views about behavioural problems observed in their daughters during adolescence

Behavioural Problems	*n*	f
Positive Behaviours		
No change in behaviour	1	1
Negative Behaviours		
Increase in negative behaviours	9	9
Giving herself physical harm	2	2
Giving harm around her	2	2
Increase in obsessions	7	7
Screaming	3	3
Annoyed	1	1
Aggressive	1	1
Nervous	2	2
Pugnacious	5	5
Energetic	2	2
**Grand Total**	**36**	**36**

(n: number of responding participants, f: repetition number of the responds)

*(M2) “…She was very aggressive, nervous and her obsession level increased.”*

*(M3) “…My daughter started to become aggressive. She started fighting with her siblings and neighbours. Sometimes, she had a crying attack. She swears. Her obsession level began to increase. We cannot calm her down.*

*(M4) “…she screams a lot and slaps herself. Her obsessions also increased.”*

*(M6) “…There was barely any change. I mean, according to me, she is always the same. She is already a problematic and sick child… She always repeats the same things. She turns around herself all the time.”*

*(M7) “She cries a lot, and she doesn’t stay still. She pulls out her hair, slaps us. She beats her siblings a lot…”*.

According to Table 5, when the views of the mothers on the person who performs the hygiene and self-care of their daughters during the menstrual period are examined, it is determined that the mothers mostly perform the hygiene and self-care of their daughters. In addition, one of the mothers stated that if she is not at home, her daughter’s elder sister helps her in this matter. Quoted statements that can be examples of mothers’ views on the person who performs hygiene and self-care for their daughters during menstruation are given below:

**Table 5:** Views of the mothers about the person performing Hygiene and Self-Care of their daughters during menstrual period

The Person Who carries out Hygiene and Self-Care	*n*	f
Mother		
Mother	10	10
Others		
Elder Sister	1	1
**Grand Total**	**11**	**11**

(n: number of responding participants, f: repetition number of the responds)

*(M1) “…mother and elder sister.”*

*(M3) “…I am doing everything all by myself alone and having a very hard time. Sometimes, we have problems. She undresses and wants to walk around naked. I am having difficulty in bathing her.”*

*(M8) “…It’s only me. She does not let anybody do it.”*

*(M10) “…It’s only me. Who else will do it? Since she attacks everybody, no one is inclined to do it.”*

According to Table 6, when the views of the mothers about the situations that tire/strain them during adolescence are examined, it is determined that mostly the daughters of these mothers have difficulties in using pads and in cleaning the hairs in the armpits and genital area and in taking a bath. Quoted statements that can be examples of the views of mothers on the situations that tire or strain their daughters during adolescence are given below:

**Table 6:** Mothers’ views about situations tiring/straining them during the menstruation period of their daughters

Tiring/Straining Situations	*n*	f
Difficulty in Teaching Hygiene		
Difficulty in using pad	6	6
**Difficulty in Teaching Self-Care**		
Difficulty in making her get dressed and undressed appropriately and in suitable environment	2	2
Difficulty in her bathing and making her bath	2	2
Difficulty in cleaning the hairs growing in her armpits and genital area	4	4
**Grand Total**	**14**	**14**

(n: number of responding participants, f: repetition number of the responds)

*(M2) “…she undresses everywhere.”*

*(M3) “…We mostly have difficulty in making her wear the pad. She always wants to take her pad off …”*.

*(M4) “…She continuously scream during pad change, and do not let us change the pad.”*

*(M6) “…I am always tired. Household chores, works related with children…They are never over.”*

*(M7) “…She does not let me have her bath, and she does not let me clean her hairs.”*

*(M9) “…My daughter is always difficult. For example, she does not let me shave her hairs. She does not change the pad.”*

## Discussion

It is emphasized that developing toilet habits, getting dressed and obeying daily routine instructions in early developmental stages are prerequisites for preparing the girls with ASD for menarche (Cummins et al., 2020). In this study, which we conducted with the aim of examining the menstrual hygiene and self-care skills of adolescent girls diagnosed with ASD, half of the mothers who participated in our study stated that their daughters use the toilet and clean after the toilet independently, but the majority of the mothers stated that their daughters could not perform basic self-care skills such as combing hair, cutting nails, brushing teeth, bathing dressing and undressing independently. Mademtzi et al. (2018) reported in their study in which they sought parental opinions that adolescent girls with ASD do not pay attention to their personal hygiene and general appearance and have poor self-care skills, which is similar to our study. Poor self-care skills are important problems for girls with ASD who already have limited social interactions, as it can create more social interaction problems and negatively affect the individual’s self-perception. Schuttler (2017) revealed that social skills and self-care of individuals with ASD could be improved in natural community settings and with the support of their peers through a self-care program designed to meet special needs.

It has been stated that mothers of individuals with ASD tend to be more concerned with basic life skills and social cohesion rather than individualism and independence (Navot et al., 2017). In this study, hygiene and self-care of girls with ASD was found to be mostly carried out by mothers, with only one mother stating that her able-bodied daughter helped to care for her sister with ASD when she was away. This situation is a barrier to independence for people with ASD. Therefore, in order for people with ASD to continue their lives and develop their social skills without being dependent on the care of others, their parents should be informed, educated and supported in delegating responsibilities to their children.

All of the mothers who participated in this study stated that nobody has informed their daughters about the adolescence period and various changes that may occur in this period, and that they have not made any preliminary preparations for the adolescence period. It was reported that individuals with ASD have experienced significant difficulties related to the physical changes that have occurred during adolescence, which is related to their resistance to change (Hellemans et al., 2010; Dewinter et al., 2013). Among the mothers participating in our study, it was revealed that only one mother told her daughter about the physical changes that occur in the body during adolescence (breast growth and hair growth in certain parts of the body), while two mothers taught their daughters that their breasts would grow during puberty, and blood may come from their vagina, and she taught her daughter how to use sanitary pads. In addition, it was determined that all mothers participating in the study did not teach their daughters how to care and clean unwanted hairs (armpit, genital area) growing during adolescence and requiring hygiene. While one of the mothers stated that she did not know what to do about informing her daughter about puberty, another mother states that her daughter did not understand what she says. Nichols and Blakeley-Smith (2009) stated in their study that some parents are willing to provide information about adolescence for their adolescent children, but they do not have enough knowledge, skills and resources to do so. As a matter of fact, in a study conducted by Anisa (2019), although similar results were revealed, she stated that teenagers with ASD should be educated on issues related to the menstrual period, and that parental counselling should be provided for mothers so that these issues can be understood better. Considering the educational status of the mothers who participated in this study, it is thought that the situation determined in the present study may be due to the low education level of the mothers or the mothers’ lack of belief that their children can succeed. In addition, considering the region where the study was conducted, it can be considered that mothers could not inform their daughters about menstruation due to cultural characteristics and religious beliefs. The reason for this is that menstruation is a frightening, worrying or embarrassing event in some societies, and that there are similar attitudes towards menstruation in Turkish society (Kırbaş, Kahriman & Kaşko-Arıcı, 2022), in the region, where this study was conducted, the definitions such as ‘getting sick’ or ‘getting dirty’ are used for menstruation period among women.

In the literature, previous studies on the subject indicate that it is difficult for girls with ASD to adapt to menstruation, hygiene and the changing body during adolescence (Navot et al., 2017). Although Cridland et al. (2014) reported that girls with ASD cope well with the changes brought about by adolescence, Steward et al. (2018) in their study with adolescent girls with and without ASD, found that girls with ASD had a particularly difficult menstrual period, and reported that they saw it as a sad and challenging event. Fei et al. stated that girls with ASD need help with menstrual hygiene and that many families are concerned about the fact that their daughters may have problems with menstrual hygiene if they are not at home during the menstrual period (Fei et al., 2021). Memarian and Mehrpisheh (2015), on the other hand, stated that parents of individuals with ASD seek medical support in order to solve the problems they encounter during adolescence, especially regarding menstruation. In this study, when the views of the mothers on the tiring/straining situations during their daughters’ adolescence are examined, it is determined that the mothers mostly have difficulties in using the pads of their daughters, cleaning the hairs in the armpit and genital area, and bathing their daughters. In addition, mothers reported that they thought that their daughters were not in a position to receive any support or ask for help regarding adolescence of their daughters. This situation can be considered as a reason why all mothers who participated in the study took such active responsibility for their daughters’ difficulties in basic self-care and menstrual hygiene.

It is known that the menstrual period has negative effects on the mood, behavioural and physical symptoms of girls with ASD as in other adolescents, and it exacerbates the problems in sensory, emotional and behavioural regulation in girls with ASD (Steward et al., 2018). According to some studies, it was revealed that the girls with ASD feel worse during menstruation and have difficulties in participating in social life (Cummins et al., 2020; Stewards et al., 2018). Fei et al. (2021) reported in their study that girls with ASD constitute the majority of patients who applied for pre-pubertal guidance, and menarche has an exacerbating effect on emotional problems and mood regulation. Although one of the mothers participating in our study stated that there was no change in her daughter’s behaviour during adolescence, rest of the other mothers reported that they mostly observed an increase in negative behaviours in their daughters during this period, and that there was an increase in their obsessions and that their daughters became more aggressive. These behavioural problems, which increase in girls during adolescence, cause stigmatization and social withdrawal of both adolescent girls and their families, creating a new problem in addition to existing problems.

## Conclusion and Suggestions

As a result of the study, it was determined that adolescent girls with diagnosed ASD could not perform their self-care skills sufficiently. It was determined that almost all of the hygiene and self-care of adolescent girls with ASD were performed by their mothers. It was concluded that the mothers of adolescent girls with ASD did not make any preliminary preparations for the adolescence period. According to the results of the study, it was determined that the situation that tire/strain the mothers most during menstruation period was generally the difficulty experienced about their daughters’ using pads, the cleaning of the hairs growing in armpits and genital area and having them bath. In addition, all mothers who participated in the study reported that their daughters’ negative and obsessive behaviors increased during this period and that their daughters became more aggressive. In accordance with these results, the following suggestions can be made:

- That the girls with ASD should be equipped with self-care skills from the early ages,
- That parents of the daughters experiencing difficulty in their self-care skills especially the mothers who are the primary person in charge of this care should be provided with education about this issue,
- That family members apart from mother such as father, elder sister, grandmother should take responsibility in equipping the adolescent girls with ASD with menstrual hygiene and self-care skills,
- That preparing psychoeducational programs for adolescent girls with ASD, giving trainings to girls and their families on the use of pads and how to clean unwanted hair by experts in the field should be provided,
- That the girls with ASD should be taught how to cope with emotional and behavioural changes that they possibly experience during adolescent period.
- That conducting studies specific to menstrual hygiene behaviours and practices of girls with autism in different cultures and regions should be provided.

### Limitations of the Study

The limitations of this study are only mothers are included in the study. The study was conducted in the city centre of Batman and the young people attended a private educational institution. Another limitation of the study is that only mothers’ opinions were included among the parents in the region where the research was conducted, and these opinions are open to bias because they are subjective. In addition, the results of this study can only be generalised to adolescent girls with ASD in the region where the study was conducted. For this reason, it is recommended that future studies should compare the menstrual hygiene of typically developing adolescent girls and adolescent girls with ASD, and that the study should be conducted with a larger sample group.
